# Examining an App-Based Mental Health Self-Care Program, IntelliCare for College Students: Single-Arm Pilot Study

**DOI:** 10.2196/21075

**Published:** 2020-10-10

**Authors:** Emily Lattie, Katherine A Cohen, Nathan Winquist, David C Mohr

**Affiliations:** 1 Department of Medical Social Sciences Northwestern University Chicago, IL United States; 2 Department of Preventive Medicine Northwestern University Chicago, IL United States; 3 Department of Psychiatry and Behavioral Sciences Northwestern University Chicago, IL United States

**Keywords:** mHealth, college students, depression, anxiety, mobile phone

## Abstract

**Background:**

In recent years, there has been an increase in symptoms of depression, anxiety, and other mental illnesses in college student populations alongside a steady rise in the demand for counseling services. Digital mental health programs, such as those delivered through mobile apps, can add to the array of available services but must be tested for usability and acceptability before implementation.

**Objective:**

This study aims to examine how students used IntelliCare for College Students over an 8-week period to examine the preliminary associations between app use and psychosocial targets and to gather user feedback about usability issues that need to be remedied before a larger implementation study.

**Methods:**

IntelliCare for College Students is an app-based platform that provides symptom assessments with personalized feedback, information about campus resources, lessons on mental health and wellness topics, and access to the suite of interactive skill–focused IntelliCare apps. A total of 20 students were recruited to participate in an 8-week study. To test for a broad range of potential users, we recruited a mixed sample of students with elevated symptoms of depression or anxiety and students without elevated symptoms. Participants completed psychosocial questionnaires at baseline, week 4, and week 8. Participants also completed user feedback interviews at weeks 4 and 8 in which they provided feedback on their experience using the app and suggestions for changes they would like to be made to the app.

**Results:**

Of the 20 students who downloaded the app, 19 completed the study, indicating a high rate of retention. Over the study period, participants completed an average of 5.85 (SD 2.1; range 1-8) symptom assessments. Significant improvements were observed in the Anxiety Literacy Questionnaire scores (Z=−2.006; *P*=.045) and in the frequency with which participants used both cognitive (Z=−2.091; *P*=.04) and behavioral (Z=−2.249; *P*=.03) coping skills. In the feedback interviews, we identified a high degree of usability with minor bugs in the app software, which were quickly fixed. Furthermore, in feedback interviews, we identified that users found the app to be convenient and appreciated the ability to use the program in short bursts of time.

**Conclusions:**

The findings indicate that the IntelliCare for College Students program was perceived as largely usable and engaging. Although the program demonstrated usability and preliminary benefits to students, further testing is needed to determine its clinical utility among college students.

**Trial Registration:**

ClinicalTrials.gov NCT04035577; https://clinicaltrials.gov/ct2/show/NCT04035577

## Introduction

Over the last decade, the number of college students experiencing symptoms of depression and anxiety has increased [1]. Students with these and other mental health concerns are typically directed toward campus counseling centers as the first, and many times only, option for treatment. Consistent with the rising rates of depression and anxiety, the number of students seeking mental health services in campus counseling centers has increased. A national survey of college students showed that 6.6% of students received treatment at a college counseling center in 2007, whereas 11.8% of students received treatment in a college counseling center in 2017 [2]. As a result, campus counseling centers often report that they were over capacity and unable to immediately meet the needs of the large number of students requesting services. Limited available sessions, long wait lists, and small staff numbers are among a number of concerns that counseling centers across the United States encounter. To address these concerns and ensure that students receive care, innovative and scalable solutions are needed.

Mobile apps and other digital mental health programs are increasingly being investigated as tools to supplement or enhance care on college campuses. Digital mental health programs offer the potential to provide self-management tools and help triage students to appropriate services on campus, thus lessening the burden that counseling centers may face from students with low-level concerns and increasing the number of students who can receive support. The efficacy of digital mental health programs in college populations has been established. A systematic review found that the majority of digital mental health interventions for college students were effective in producing changes in psychological outcomes, such as depression and anxiety, yet few of the tested programs were publicly available and fewer were delivered via apps [3]. In addition, the vast majority of college students own smartphones, making apps a potentially accessible option to deliver services [4]. In one study, although a small number of students had used a mental health app, more than one-fourth were open to using an app. At the same time, students were largely unsure of whether mental health apps were evidence based and voiced concerns about the efficacy and impersonal nature of apps [5]. This suggests that acceptability is a key factor to investigate when researching mental health apps for college students; students may find apps with clear research evidence or apps tailored to their specific needs more acceptable.

The IntelliCare app platform has demonstrated efficacy in reducing symptoms of depression and anxiety in general adult populations [6-8]. IntelliCare is a collection of quick and easy-to-use apps that each present a different evidence-based mood management strategy (eg, cognitive restructuring, behavioral activation). Although typically deployed in conjunction with coaching support delivered via text message, the results of a recent trial indicate that the effect of coaching was low and that users who did not receive coaching experienced similar benefits in reducing depression and anxiety symptoms. Although human support in the form of coaching has been shown to be effective for engaging users in mobile health interventions, it is also costly, and many colleges are working with very limited budgets for mental health care. Fully automated interventions are nonconsumable resources in that they can benefit a broad array of individuals without requiring additional therapeutic power. Thus, the cost is less dependent on the number of individuals accessing the intervention [9]. Thus, as we developed a version of IntelliCare specifically for college campuses, we opted to eliminate coaching so that the intervention could be used independently by students and be maximally scalable. The adaptation of IntelliCare to form the IntelliCare for College Students program was guided by a series of user-centered design activities with college students and counseling center staff members [10,11].

In this paper, we present an extended usability pilot study of the IntelliCare for College Students app that was conducted in preparation for the implementation of the app on 2 university campuses. The study was not powered nor intended to evaluate clinical outcomes, but rather, the purpose of this study is to examine how students used the app over an 8-week period, to examine the preliminary associations between app use and psychosocial targets, and to gather user feedback about usability issues to be remedied before the larger implementation study.

## Methods

### Recruitment

Students were recruited from 2 public 4-year universities in the same Midwestern state. Both universities involved in this study have large student bodies and serve more than 10,000 students per year. Print advertisements were posted in multiple buildings on the universities’ campuses with information regarding *IntelliCare for College Students* and the research study. Digital recruitment materials were spread through social media and sent in mass emails to students. Research staff contacted student organizations and campus offices at each university to assist in disseminating recruitment materials to students. Interested students completed a screening questionnaire to determine eligibility for the study that included questions on demographics, student status, smartphone ownership, and symptoms of depression and anxiety. For screening purposes, symptoms of depression and anxiety were assessed using the nine-item Patient Health Questionnaire (PHQ-9) [12] and Generalized Anxiety Disorder seven-item scale (GAD-7) [13]. Scores on the PHQ-9 can range from 0 to 27, and scores on the GAD-7 can range from 0 to 21. To ensure a range of symptoms of depression and anxiety, we attempted to recruit a balanced sample of participants who had *higher symptoms* (defined as a baseline PHQ-9 or GAD-7 score ≥10, indicating at least moderate symptoms) and those who had *lower symptoms* (defined as baseline PHQ-9 and GAD-7 scores both <10) from each university. Eligible students were required to (1) be enrolled either part time or full time at one of the universities studied, (2) be at least 18 years old, (3) own an Android smartphone capable of running version 7.0 or higher or own an iPhone capable of running iOS 11 or higher, and (4) fill in a spot in either the higher symptom group or lower symptom group. Of the eligible participants who downloaded the app, 11 were in the higher symptom group and 9 were in the lower symptom group. A sample size of 20 was established a priori based on previous data, suggesting that 20 users can find 95% of usability problems [14].

### Intervention

Eligible participants (n=20) were instructed to download the *IntelliCare for College Students* app and encouraged to use it as they desired for 8 weeks. Several features were included in the *IntelliCare for College Students* app: (1) a mood rating and mood journal tool that allowed participants to rate their mood using an emoji-based scale and subsequently write a few sentences about their mood; (2) a calendar tool that allowed participants to access a history of their mood rating and mood journal entries; (3) a weekly symptom check that provided participants with personalized feedback, such as advice on managing stress and connecting with others when symptoms were elevated; (4) information about on-campus resources specific to participants’ university along with links directing students to websites with more information; (5) short psychoeducational lessons on mental health and wellness topics (eg, *Self Care 101*); and (6) the suite of interactive skill–focused IntelliCare apps that were available for download. Screenshots of each of these features are available in Multimedia Appendix 1.

### Measures

Participants were prompted to complete the PHQ-8 [15] and GAD-7 [13] on a weekly basis as part of the symptom check. The PHQ-8 was selected as the symptom check tool because the IntelliCare for College Students app was designed as a self-guided resource that, following this initial study, would be made freely available to all interested students at these universities. Although it is established that asking about suicide via self-report questionnaires does not increase the risk of suicidal thoughts or behavior, university administrators voiced a strong preference to avoid asking about suicidality within the app because it would not be possible to closely monitor the responses in the broader implementation of the program. At baseline, week 4, and week 8, participants completed the *Check my Knowledge Questionnaire* through the app, which included the Anxiety Literacy Questionnaire (ALQ) [16], Depression Literacy Questionnaire (DLQ) [17], Knowledge and Beliefs about Services Scale (KBSS) [18], and Cognitive and Behavioral Response to Stress Scale (CB-RSS) [19]. The ALQ and DLQ are designed to assess mental health literacy by presenting 22 true or false statements regarding anxiety and depression (eg, *Being easily fatigued may be a symptom of anxiety disorder*). Participants receive a score of 1 for each statement they correctly assign as either true or false. Scores for each questionnaire can range from 0 to 22, with higher scores indicating greater mental health literacy. The KBSS includes 5 questions designed to measure knowledge of campus mental health services (eg, “What have you heard from other students about the quality of mental health and psychological counseling services on your campus?”). Responses to each item on this measure are independently examined. The CB-RSS is an 18-item scale designed to measure the use and helpfulness of various cognitive and behavioral skills. There are 4 subscales included: cognitive skill frequency, cognitive skill usefulness, behavioral skill frequency, and behavioral skill usefulness. For each cognitive or behavioral skill, participants rate how often they used the skill and how helpful it was (eg, “During the past month, how often did you take a moment to notice things that made you feel good or grateful? How helpful was this in making you feel better?”). Scores for cognitive subscales range from 0 to 24, and scores for behavioral subscales range from 0 to 30.

At weeks 4 and 8, the participants completed 30-min semistructured user feedback interviews. Participants provided feedback on their experience using the app and suggestions for changes they would like to see be made to the app (example questions include, “What problems have you encountered using IntelliCare for College Students?” and “What changes would you make to IntelliCare for College Students?”). Interviews were audio recorded and conducted via telephone.

Participants were eligible to receive a total of US $70 for participating in the study, including US $10 for completing each of the monthly in-app assessments and US $20 for completing each of the user feedback interviews.

### Data Analysis

We incorporated quantitative data from questionnaires and app usage logs and qualitative data from interviews for a mixed methods data analysis. This mixed methods approach was chosen because although quantitative data can identify usability issues and dissatisfaction with program components, qualitative data provide guidance to the root of those errors and methods for program optimization. App usage data were examined in the form of descriptive statistics. We recruited a balanced sample of participants who had *higher symptoms* (defined as a baseline PHQ-9 or GAD-7 score ≥10) and those who had *lower symptoms* (defined as baseline PHQ-9 and GAD-7 scores both <10). Owing to the small sample size of each group, we examined symptoms of depression and anxiety by subgroup in the form of descriptive statistics. Measures of our treatment targets (anxiety literacy, depression literacy, and cognitive and behavioral coping skills) were analyzed using Wilcoxon signed-rank tests across participants with higher symptoms and those with lower symptoms [20]. Qualitative data were analyzed using a thematic analytic approach [21] in which interviews were analyzed using iterative codes that were used to identify core concepts, from which we determined the needs, concerns, and impressions of our program.

## Results

### Participants

A total of 30 people initiated the web-based screening questionnaire. Overall, 2 potential participants did not proceed with the full screening questionnaire, one potential participant was ineligible because they were not a student, and the remaining 6 participants were ineligible because we had closed recruitment for their cluster (eg, University #1, higher symptom). Our target recruitment goal was 20 participants. Of the recruited participants, 1 withdrew from the study before installing the study app, and we enrolled an additional participant in her place, leaving us with a final sample of 21 consented students and 20 students who installed the study app and initiated the study procedures.

Of the total sample of consented participants (n=21), 11 (52%) participants identified as White, 4 (19%) participants as African American or Black, 4 (19%) participants as Asian, and 2 (9%) participants declined to report their racial identity. Moreover, 28% (6/21) participants indicated that they were more than one race. The mean age of the participants was 24.19 (SD 6.03) years. The majority of the sample was female (n=17), non-Hispanic (n=17), and seeking undergraduate degrees (n=14). Of the final sample (n=20), 19 participants completed the study, indicating a high rate of retention.

Of the total sample, 11 participants reported elevated symptoms for depression and/or anxiety (defined as a PHQ-9 or GAD-7 score ≥10), and we refer to this group of participants as the *higher symptom participants*. Of those participants, 6 reported elevated symptoms of both depression and anxiety. Participants without elevated symptoms of depression or anxiety are referred to as the *lower symptom participants*.

The average PHQ-9 score for participants with higher symptoms was 13, indicating moderate symptoms of depression (SD 5.63; range 7-27), and the average PHQ-9 score for participants with lower symptoms was 3.60, indicating few symptoms of depression (SD 2.59; range 0-8). The average GAD-7 score for participants with higher symptoms was 12.35, indicating moderate symptoms of anxiety (SD 4.74; range 7-21), and the average GAD-7 score for participants with lower symptoms was 3.50, indicating few symptoms of anxiety (SD 3.37; range 0-9).

### App Usage

Across the sample, the IntelliCare for College Students program was used an average of 17.05 days over the 8-week study period (SD 8.12; range 4-25). Participants completed an average of 5.85 symptom assessments (SD 2.1; range 1-8). In examining the time between the first and last use of the app, we saw that the majority of participants (18/20, 90%) continued to use the app beyond the 8-week study period. Of the 2 participants who did not use the app beyond the 8-week study period, there was a 9-day period of use for 1 participant and a 42-day period of use for the other participant.

There were minimal differences in usage between students with higher symptoms and those with lower symptoms. Participants with higher symptoms used it an average of 18.91 (SD 9.83) days, and participants with lower symptoms used it an average of 14.78 (SD 5.02) days. Participants with higher symptoms completed an average of 6.09 symptom assessments (range 1-8), and participants with lower symptoms completed an average of 5.55 symptom assessments (range 2-8). Moreover, 1 participant from each group did not use the app beyond the 8-week study period.

### Psychosocial Targets

In the full sample, significant improvements were observed in participants’ scores on the ALQ (Z=−2.006; *P*=.045). We also observed significant increases in the frequency with which participants used both cognitive (Z=−2.091; *P*=.04) and behavioral (Z=−2.249; *P*=.03) coping skills, as measured by the CB-RSS. Significant changes were not observed for the DLQ (*P*=.23) or for the perceived usefulness of cognitive (*P*=.06) and behavioral coping skills (*P*=.09). Table 1 provides descriptive statistics on psychosocial targets for the full sample and by subgroup.

Although this study was not powered to detect differences between the participants with higher symptoms and lower symptoms, it appeared that the statistically significant improvements observed in the ALQ and the frequency subscales of the CB-RSS were driven by participants in the higher symptom group. Participants in the higher symptom group began the study with lower scores and at week 8 had scores more similar to those in the lower symptom group.

Minimal changes were observed over time for items on the KBSS. At baseline, 11 participants agreed or strongly agreed that they knew where to go if they needed to seek professional help for their mental health while attending their university, and at the 8-week follow-up, 13 participants agreed or strongly agreed with that statement. At baseline, nearly all participants (n=18) responded that they believed that therapy or counseling is *very helpful* or *quite helpful* for individuals their age who are clinically depressed, and this did not change at 8-week follow-up.

No meaningful changes were observed in the PHQ-8 or GAD-7 scores over time in either subgroup. At the week 1 symptom check, participants in the higher symptom group had a mean score of 11.63 (SD 6) on the PHQ-8 and a mean score of 10.82 (SD 4.12) on the GAD-7. By the week 8 symptom check, participants in the higher symptom group had a mean score of 9 (SD 6.53) on the PHQ-8 and a mean score of 10 (SD 3.83) on the GAD-7. Similarly, at the week 1 symptom check, participants in the lower symptom group had a mean score of 3.75 (SD 2.38) on the PHQ-8 and a mean score of 4.88 (SD 2.85) on the GAD-7. By the week 8 symptom check, participants in the lower symptom group had a mean score of 0.6 (SD 2.51) on the PHQ-8 and a mean score of 2.86 (SD 2.61) on the GAD-7.

**Table 1:** Descriptive statistics of psychosocial targets for the full sample and by subgroup.

Criteria	Full sample, mean (SD)		Higher symptom, mean (SD)			Lower symptom, mean (SD)		
	Baseline	Week 8		Baseline	Week 8		Baseline	Week 8
Anxiety Literacy Questionnaire	13.88 (3.67)	14.63 (3.45)		13.6 (3.91)	14.4 (3.97)		14.25 (3.62)	14.89 (2.98)
Depression Literacy Questionnaire	13.89 (4.26)	14.68 (3.83)		13.6 (3.34)	14.7 (3.68)		14.22 (5.31)	14.67 (4.21)
CB-RSS^a^ cognitive usefulness	9.73 (6.23)	12.58 (6.86)		8.4 (6.24)	12.6 (7.17)		11.22 (6.22)	12.56 (6.93)
CB-RSS cognitive frequency	10.47 (4.03)	12.68 (4.49)		9.6 (4.81)	13.4 (5.02)		11.44 (2.92)	11.89 (3.98)
CB-RSS behavioral usefulness	17.74 (6.10)	19.68 (8.91)		16.2 (6.09)	18.6 (9.98)		19.44 (5.98)	20.89 (7.98)
CB-RSS behavioral frequency	14.42 (4.98)	16.52 (6.44)		13.9 (5.61)	16.5 (6.72)		15.0 (4.44)	16.56 (6.54)

^a^CB-RSS: Cognitive and Behavioral Response to Stress Scale*.*

### Qualitative User Feedback

A total of 3 main themes were identified from student feedback on their experiences with the IntelliCare for College Students program: opportunities for self-reflection, access to information and resources, and convenience.

#### Opportunities for Self-Reflection

Students enjoyed having the space to write down how they were feeling and found the process of having an emotional check-in spot in the form of a mood journal, particularly useful. One student noted:

It’s the first time I’ve ever used an app...to say what I’m feeling. I’ve never attempted to use apps or basically tell anyone how I feel [on] a certain day...But, actually like even typing it down, it feels kind of...relieving in some way.

This highlights that the act of reflecting on and verbalizing one’s emotional experiences was perceived as beneficial for stress management and self-care.

There was a similar appreciation voiced for the symptom checker, which guides users through an assessment of common depression and anxiety symptoms. As one student noted:

So I was, because I don’t go for counseling and all that because I don’t think I have super severe problems but...I have my days where I’m done and I think it’s critically important for people to check in with themselves sometimes because most people don’t do that often...it’s kind of nice to open and app and, “How are you doing?” and “Do you feel this? Do you feel that?” it’s like a check in kind of thing.

Here, we observe that the app was seen as a way to check in with oneself and to prompt users to take note of specific symptoms and emotional experiences that they might be having and otherwise not be aware of.

Many students were interested in tracking their symptoms and connecting those symptoms to potential stressors and/or triggers. As one student commented:

I think what motivates me is to track my moods in general is just finding those connections with what I wrote and the things going on in my life.

Students reflected on their process of self-discovery through using the mood journal, noting that they identified differences in how they felt based on place (eg, school vs home) and time (eg, different times of the month) and were able to use those identified differences to enact changes in their lives.

#### Access to Information and Resources

Students also valued being able to access information about mental health and stress through the IntelliCare for College Students app. When prompted about their experiences using the app, one student reflected that the app contained:

some useful information, like about depression and anxiety. Yeah some information that I wasn’t familiar with. So yeah, maybe it gave me a better understanding of like some terms or maybe like some symptoms.

Although the information included in IntelliCare for College Students app was not entirely novel to some participants, the consolidated delivery format and the ability for students to access this type of information conveniently within the app were seen as the strength of the program. One student reflected:

So, I like this because I think different at this point is so much better because even me as a Freshman we hear the same things like “Go to this” and “Go to that.” And sometimes we just don’t want to do it because we hear it so much and it takes so much effort even though sometimes it doesn’t take a lot of effort but to many of us, it does. So much work, I have to go to the building, I have to call and schedule but this is like, this would be a good first step for many people and you don’t have to be someone who’s, who has depression or anybody, you could, it could work for people just as a self-check because checking in with yourself and how you handle situations is important.

Here, we observe the multiple barriers that the student had faced to go to the counseling center and how she saw the app as a good entry point for any student to check in on their mental health status and learn about the resources available to them.

#### Convenience

Mobile mental health apps have historically been developed to increase convenient access to mental health information and tools, and convenience was a major theme in the user feedback interviews. Students indicated that they appreciated the ability to use the program in short bursts of time. One student noted that she was able to engage with the app more continuously because of this design, as in:

It’s like having a diary without having to do all of the writing. Because I was one of those people where I had a diary and I’d use it for like two days and then I’d stop. But it’s...doing the work without putting in so much effort. So you’re just answering general questions like ‘Are you feeling this? Have you been feeling this?’ I like that idea behind it because it’s not super time-consuming.

Another student commented:

I feel like when I use it, I don’t really spend a lot of time on there, which is a good thing because it’s very straight-forward too so I kinda know which app to open or like whatever needs I’m having that day. So it’s really user-friendly.

Similar comments highlight that the program was perceived as usable and convenient for students.

However, although students generally agreed that the program was quick and easy to use, many students identified a combination of lack of time and forgetfulness as barriers to program use. One student commented:

When I have a lot going on, the first thing on my mind isn’t IntelliCare...So, these last 4 weeks, that’s when my classes started. And so it was kind of hard to like, post something when I have so many other things to do. It kind of slips my mind sometimes.

Although interactions within the app were relatively brief, several students noted that they deprioritized using the app when they were feeling strapped for time.

As one student commented:

Yeah, because honestly this really wasn’t for me, or up to me, and if I had more time in my life I would probably like schedule out like a time and use it every single day. But, like because I have so much to do and it really gets in the way of that.

When prompted about what got in the way of using IntelliCare for College Students, a student commented*:*

I’m mostly just like busy and forget like, “Oh, I have that.” Because it’s like on my phone and there’s like so much stuff on my phone already.

Although students noted that they spend a lot of time on their phones, the app often got lost in their long lists of other downloaded and infrequently used apps.

Through the course of this study, software bugs and glitches were identified, which were remedied either during or following the study. There were intermittent issues with questionnaires being deployed at appropriate intervals and with the user interface displaying and functioning properly on phones with various screen sizes. Of note, we learned that the in-app notifications were working inconsistently for our participants. We observed that many students valued the use of notifications to address issues related to forgetfulness. One student who received notifications throughout the study commented:

I feel like it’s good to have um, a reminder about just kind of like to check in with myself basically. That it has the notifications and I like it. You know, I have to do the check in. I feel like it’s been helping me on that. Like scheduled just thinking about my week.

Another student, whose notifications were working inconsistently, noted that they put reminders in their own personal calendar on their phone to use the app, noting:

Like I do a lot of things and I’m also a full-time student and I work full time...So I, I, that’s why I put reminders on my phone to use it but because I am so busy, like I only find myself going to it when I’m really going through a hard time.

Although forgetfulness was a notable barrier for many students and notifications were desired and useful, receiving notifications did not prompt all students to engage with the app. Rather, there needed to be a perceived need for using IntelliCare (eg, *going through a hard time*) coupled with remembering that IntelliCare was an available option (eg, receiving a notification).

Although there was generally positive feedback around the format of the IntelliCare for College Students program, ideas for enhancing the convenience and accessibility of program use were shared in the feedback interviews. Specifically, some students were interested in incorporating less text and more videos in the lessons. As one student noted:

I read through quickly two of ‘em...I feel like they should be videos recorded. But like, you know it’s all just like reading, like reading and reading. Maybe just like a video, like someone just talking to you.

Furthermore, a student noted that they might appreciate “maybe like an auditory style where it reads it to you.” Although the decision to design lessons as text based was informed by privacy considerations identified in early user-centered design activities (eg, students are often in public spaces or shared living spaces and reading allows for more discrete access to potentially sensitive mental health information), this feedback highlighted that individual students have varying preferences on how to consume information. Future iterations of the program may examine user engagement with and feedback on information presented in text, audio, and audio-visual formats.

## Discussion

### Principal Findings

Given the ongoing challenges to delivering an array of mental health services on college campuses, there remains a need to use innovative solutions to address increasing college student mental health needs. In this pilot study of the IntelliCare for College Students program, we are provided with a glimpse into what seems to work, what issues come up, and what needs to be studied next when designing and disseminating digital tools for college student mental health.

First, the IntelliCare for College Students app was considered usable and engaging. Although there were suggestions made to improve usability and there were some bugs and glitches identified during the study, the vast majority of participants (18/20, 90%) continued to use the app beyond the 8-week study period. In past trials of the IntelliCare platform for general adult populations, participants used the app beyond the 8-week study period at lower or similar rates (33.7% in one trial [6] and 84% in another trial [7]). This is particularly notable, given that mental health apps are frequently abandoned by users in a relatively short time frame. Although usage rates vary, in a review of real-world user engagement with 93 different mental health apps, Baumel et al [22] found that the median 30-day retention rates were just 3.9%. Thus, the usage rates observed in this study indicate that the program was engaging, as participants returned to the app beyond the period they had originally committed to the study.

There are often challenges in designing universally accessible mental health resources that can be used by individuals who are currently experiencing symptoms of mental illness and by individuals seeking support to promote their continued wellness. Individuals often prefer tools and resources that are perceived as personally relevant, and the tools and resources that appear personally relevant may be quite different for someone who is struggling with more significant symptoms of depression or anxiety than for someone who is looking to promote wellness through stress management practices. As individuals can fluctuate in their membership into these higher symptom and lower symptom groups over time, it is valuable to design tools that can reach individuals when they are highly symptomatic and distressed and can also support individuals when they are less symptomatic and well [23]. In our program, participants received feedback on their symptoms when they completed the symptom checker tool within the app, and we recognize that receiving feedback on symptoms could influence use. However, we observed minimal differences in usage between students with higher symptoms and those with lower symptoms, indicating that IntelliCare for College Students could be a broadly accessible program. By offering a simple workflow containing a wide variety of tools and resources for supporting mental health and wellness, participants were able to find the tools and resources that were personally relevant to them and continue to use them as needed.

We did not observe changes in symptoms of depression or anxiety during the course of this study. We note that the study was underpowered to detect changes in psychosocial targets, so we are unable to determine if the lack of change was because of chance. Compared with previous studies of IntelliCare in which participants were encouraged to use IntelliCare daily, participants in this study were not given this instruction and used IntelliCare less frequently during the 8-week study than had been observed in these previous studies [6-8]. Participants enrolled in this study during the summer months, and the 8-week follow-up for many participants took place after the fall semester began. Some fluctuations in mood may be attributed to the change from summer break to resuming coursework [24]. However, we observed improvements in psychological targets, suggesting that the program had its intended effects on behavior and condition. Improvements were observed in participants’ scores on the ALQ, indicating that they experienced gains in their knowledge of anxiety symptoms and anxiety management strategies. As low mental health literacy is a commonly cited barrier to mental health treatment seeking among college students [25,26], increasing mental health literacy appears to be a particularly important pathway to support students in seeking appropriate mental health resources.

We also observed increases in the frequency with which participants reported using a variety of cognitive and behavioral coping skills, such as taking time to figure out how thoughts impacted emotions and planning positive activities. As the use of cognitive and behavioral coping skills is believed to lead to improvements in mood [27], this finding provides preliminary support for the effectiveness of IntelliCare for College Students as a mood management resource. Significant improvements were observed in the ALQ, and the frequency subscales of the CB-RSS appeared to be driven by participants in the higher symptom group, who had lower baseline scores on these measures than did participants in the lower symptom group. Future research will examine these potential differences in larger sample sizes.

### Limitations and Future Directions

There are limitations to this study that must be considered when interpreting the results. First, this was a single-arm study design aimed at examining how students used the app over an 8-week period, the preliminary associations between app use and psychosocial targets, and usability issues that needed to be remedied before the larger implementation study. There was no control group, and the study was not powered to detect changes in psychosocial targets. Although the study sample was racially and ethnically diverse, the majority of participants were female. Although a predominantly female user base is common in studies of mental health apps [28,29] and depression is more commonly diagnosed in women than in men [24], it is unclear how generalizable the results would be to male students and additional design considerations may be needed to attract and engage a male user base. We also observed that our study sample consisted primarily of students who were already involved in their campus community through student leadership positions and past engagement in counseling services. This likely contributed to the relatively high rates of knowledge of where to seek mental health services in this sample as well as the lack of change in that knowledge over the 8-week study. Finally, because participants had committed to an 8-week study period, usage rates may have been higher than would have been in a more naturalistic study design [22]. We plan to examine and compare usage rates within this pilot study to those observed in the implementation of the IntelliCare for College Students app on 2 university campuses.

### Conclusions

The results indicate that the IntelliCare for College Students program was perceived as largely usable and engaging to a sample of university students. The program demonstrated some preliminary psychosocial benefits to students, and further testing is needed to determine the program’s utility in promoting symptom change and connecting students to other mental health resources.

## Appendix

Multimedia Appendix 1 Screenshots of IntelliCare for college students.

## Abbreviations

ALQ: Anxiety Literacy Questionnaire
CB-RSS: Cognitive and Behavioral Response to Stress Scale
DLQ: Depression Literacy Questionnaire
GAD: Generalized Anxiety Disorder
KBSS: Knowledge and Beliefs about Services Scale
PHQ: Patient Health Questionnaire
