# Supplementary figures and images for: Examining an App-Based Mental Health Self-Care Program, IntelliCare for College Students: Single-Arm Pilot Study
Source: JMIR Ment Health. 2020 Oct 10;7(10):e21075. doi: 10.2196/21075 (PMC7585772; doi:10.2196/21075)

Multimedia Appendix 1


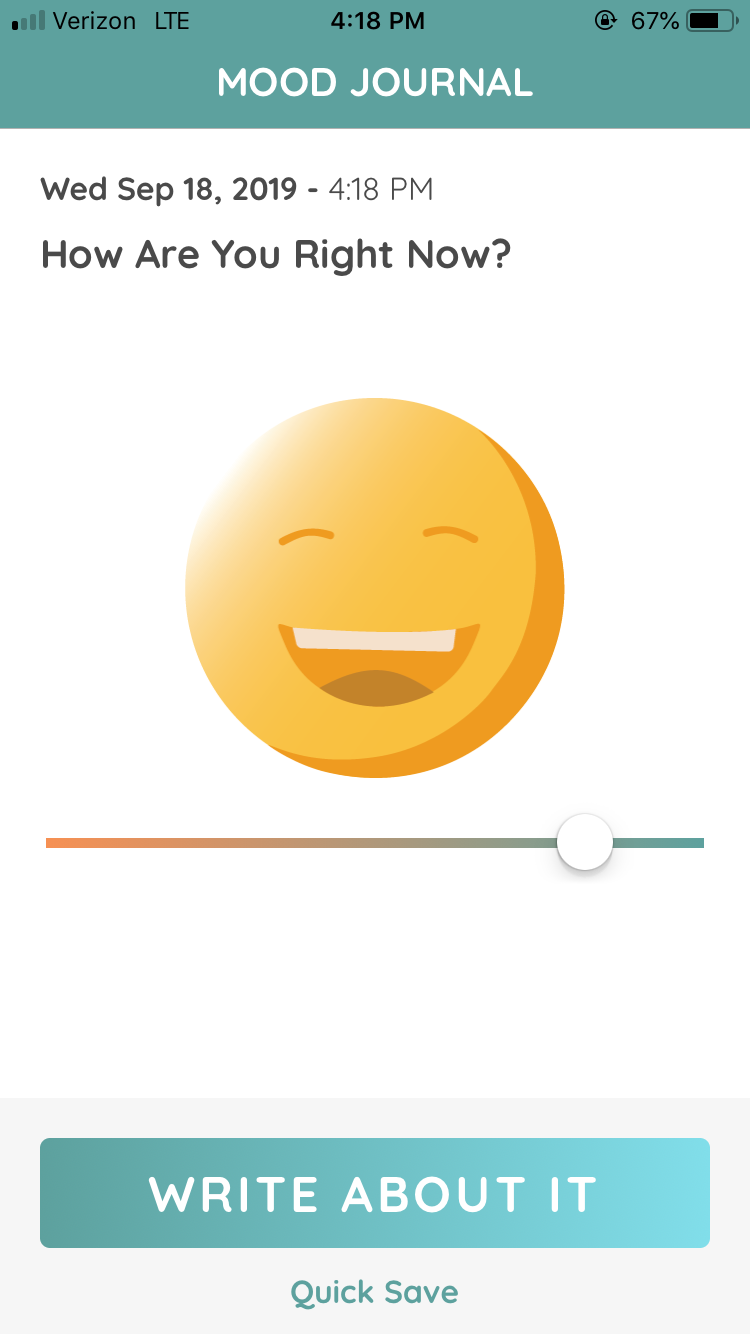

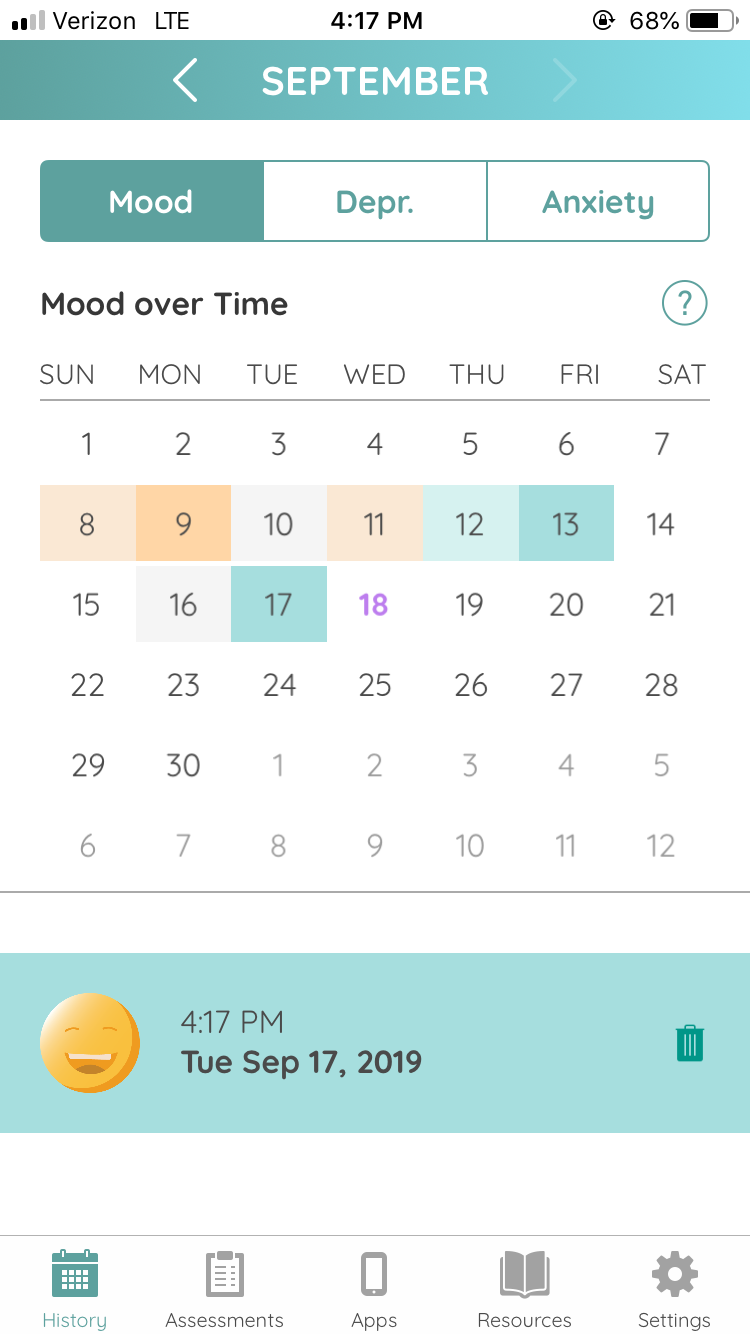

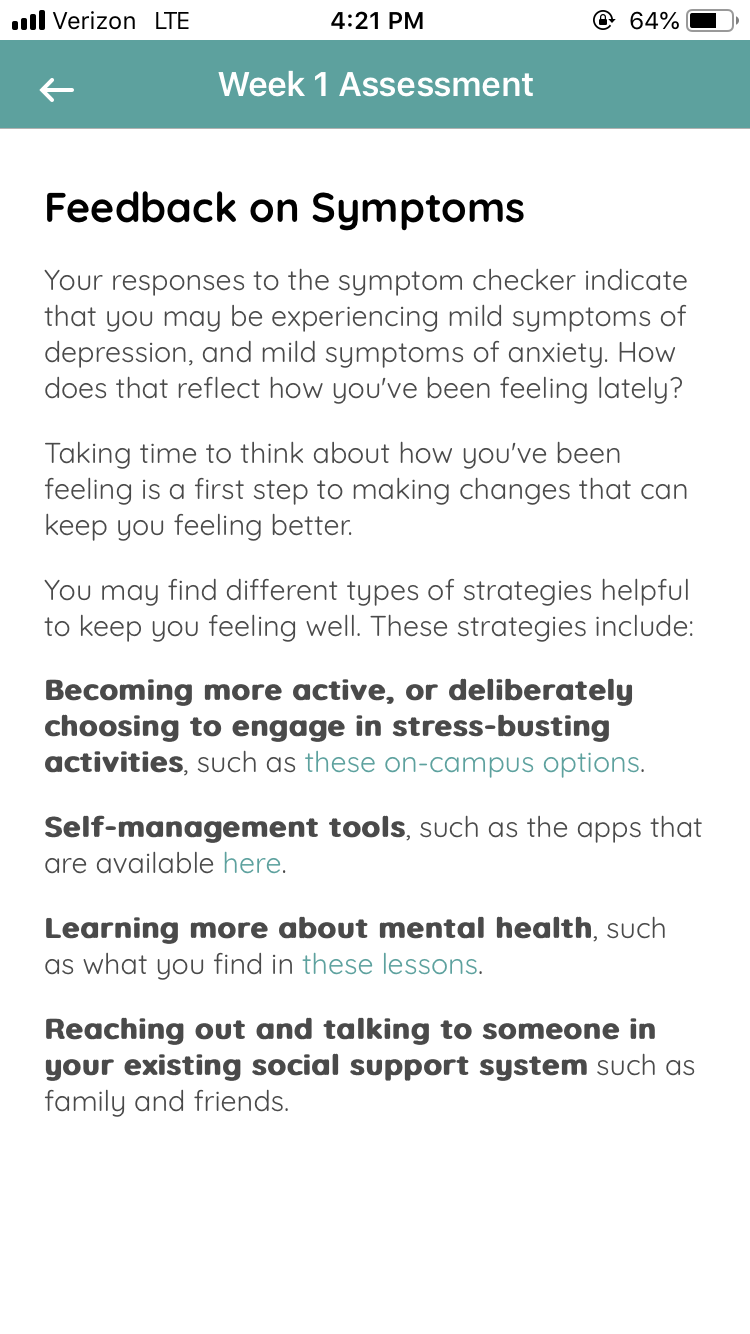


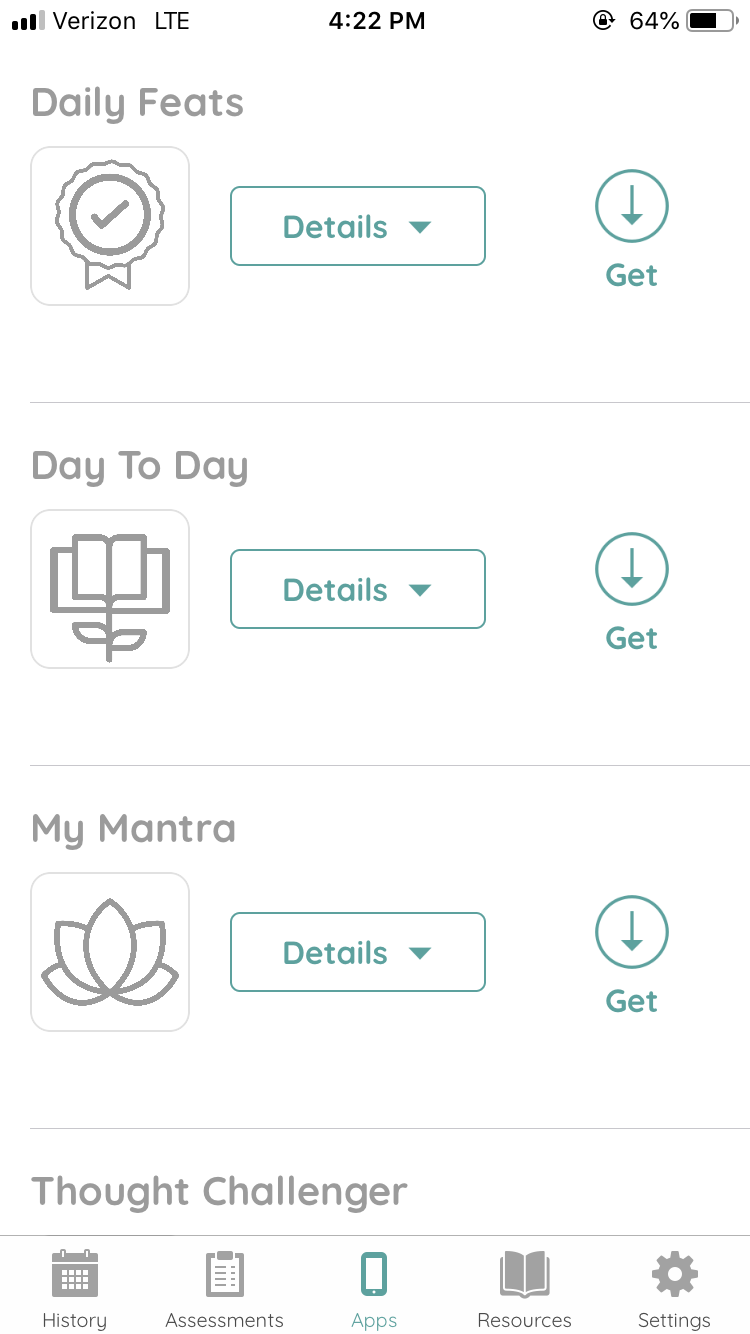

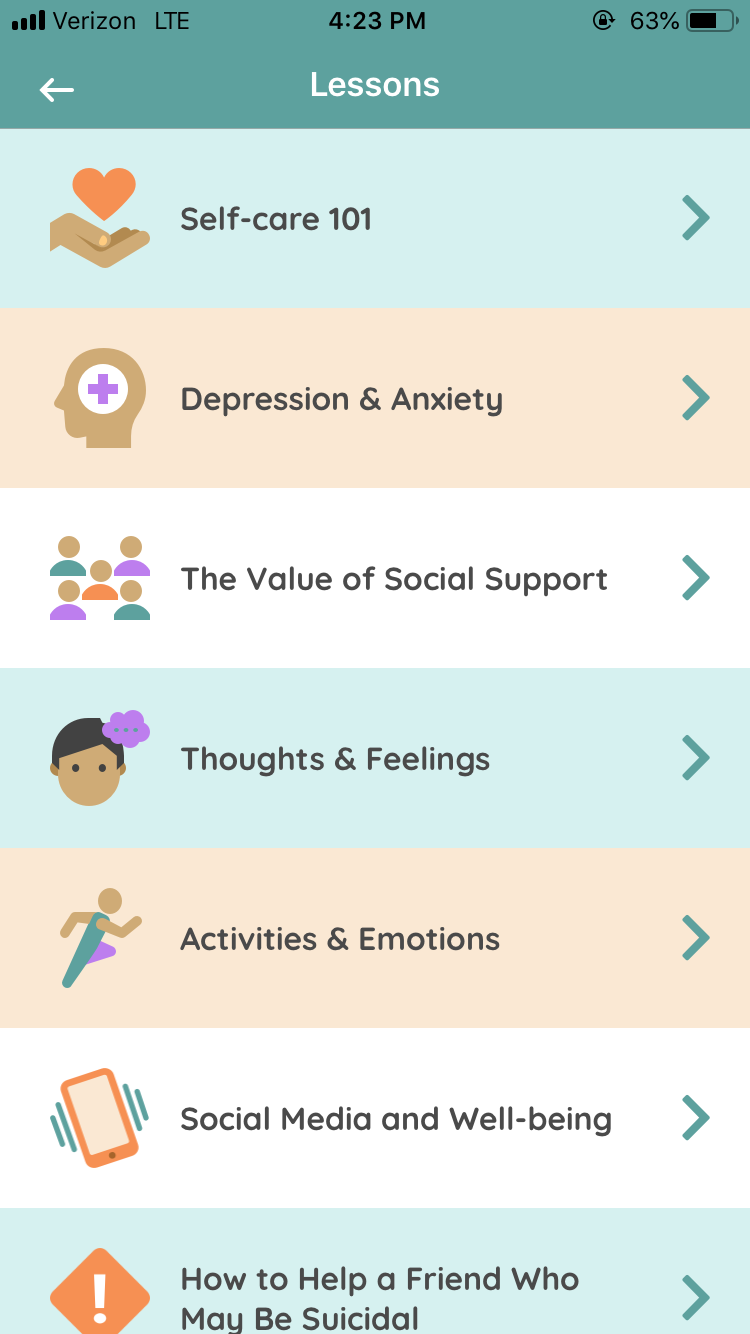

Supplement: Multimedia Appendix 1 [file mental_v7i10e21075_app1.docx]
